# My First Case of Puerperal Convulsions

**Published:** 1896-12

**Authors:** John B. Cross

**Affiliations:** Roane College, Tenn.


### MY FIRST CASE OF PUERPERAL CONVULSIONS.

ROANE COLLEGE, TENN., October 22, 1896.

On the night of October 6, 1896, I was called to see a young lady in her first confinement. On arriving at the bedside of the patient, I made inquiry as to her general health during her term of pregnancy. She informed me that her health had been very bad—bowels acted every two weeks, urine scanty, appetite poor, tongue coated brown and dry. I noticed that her hands and face were swollen considerably. On further inquiry I learned that for two months previous to her confinement she was unable to rest any at night, could not assume a recumbent position at all, complained of shortness of breath, lower extremities swollen so much she could not wear shoes. Two weeks before confinement there was rupture of right leg with a flow of liquid from opening. Eighteen months prior to confinement she had a slight stroke of paralysis of the left side with a recurrence in February last of same side. She said she was eight months pregnant.

I then endeavored, by abdominal palpation, to make out the position of the fetus, and if there was more than one, but gained nothing of importance. After thoroughly cleansing my hands, I made a digital examination per vaginam. Vertex presentation, pelvis roomy, right labia majoria swollen considerably, labor progressed nicely. Once I noticed, after a very hard pain, a rigor seemed to pass over body, yawned once, mouth slightly drawn to one side. I charged my hypodermic syringe with eight mins. tincture veratrum viride (Norwood's) in case of an emergency, gave fifteen grains each chloral hyd. and sodium bromide, used chloroform during height of pains. In a very short while she gave birth to a very small female child with single hair lip and cleft palate. In exactly twenty minutes from birth of first infant she was delivered of a second well developed child. Uterus contracted nicely, placentas and membranes came away naturally.

In three hours from birth of last child left patient to return at 4 o'clock P. M. Delivery was at 5 o'clock A. M., October 7th. Prescribed a diuretic and laxative before leaving, returned at 4 P. M., found patient doing well, taking nourishment, pulse 74 per minute, temperature normal; put on binder and left patient comfortable. At 5 o'clock P. M. she asked her husband to assist her to turn on her side. It was then she had her first convulsion, twelve hours from birth of babies. I was then five miles away. When I reached my patient the second convulsion had come on. I immediately gave her 8 mins. veratrum viride hypodermatically, repeating in twenty minutes. As soon as convulsion passed off I gave 15 grs. each chloral hyd. and bromide potass. She then commenced vomiting and continued to vomit until the last convulsion, which was nine in all. I gave an enema of chloral, while I endeavored to control the vomiting. Called another physician; did not change treatment; used chloroform by inhalation; also amyl. nitrate.

We decided not to bleed, as she was very anemic. The consulting physician gave up all hope and left. The mother of the patient asked permission to call in her family doctor. The first thing he suggested was venesection. I begged to be excused under the circumstances. He and her mother insisted, as bleeding had saved her life when she had had puerperal convulsions. So they bled in both arms, and succeeded in getting about three ounces of very black blood, which coagulated readily. Up to the time of bleeding she had had seven convulsions, each one more severe than the preceding one. I then gave her hypodermatically 4 mins. flu. ext. gelseminum, $\frac{1}{4}$ gr. morph. sulph.; had one more convulsion, gave gelseminum 6 min., $\frac{1}{4}$ gr. morph. sulph. internally; one more convulsion, the last one she had; slept four or five hours. Last convulsion 8th October. On the 9th swelling had left face and limbs to some extent, but seemed to be accumulating in abdomen, interfering with breathing a little. There was a very troublesome cough with rusty expectoration on the 10th. Breathing very difficult, had to sit propped up in bed, lips blue. I had been administering mild cathartics and diuretics all along. Discharges natural, sufficient pulse, ranging from 100 to 104 per minute, feeble, temperature 100° F. to 102° F. Case became alarming evening of the 10th inst. I called two able physicians with the idea of resorting to surgical means to get rid of abdominal accumulations, but declined until a future day, owing to feeble circulation. Prescribed cathartics, causing watery evacuations, and as severe as the case would admit. Diuretics and diaphoretics were given with excellent results. Patient did extra well; sat up on tenth day after delivery for the arrangement of bedding and toilet; also on the eleventh day. In the evening of the eleventh day temperature 104° F., pulse 108 to 112, full and strong; called for water; on attempting to drink, her tongue became stiff, uttered three or four violent screams, fell back in an unconscious condition, from which she never rallied, dying on the 14th day after confinement, face flushed, pupils dilated, pulse from 108 to 112, full and strong, temperature ranging from 102° F. to 104° F.

I think it unnecessary to give full line of treatment that was followed. I will say that I gave ammonia carbonate in five-grain doses hourly, with the idea of preventing, as much as possible, embolism or trombosis.

This is to me a very interesting case, and one, I am sure, which will never be erased from my mind.

John B. Cross, M.D.

---

### Glycerin in Certain Affections of the Stomach.

In a recent article, Sir James Sawyer remarks that some years ago Dr. Sydney Ringer recommended the administration of glycerin by the mouth in certain affections of the stomach. Acting upon his suggestion, he has since treated many cases of painful gastric digestion, such as are usually attributed to subacute or chronic catarrh of the gastric mucous membrane, with glycerin with satisfactory results. So far as he has seen, this employment of glycerin is not widely extended in professional practice, and he has not noticed further reference to it in the periodicals. The familiar routine seems to be a ringing of changes upon bismuth, alkalies, acids, and digestives. Many cases of gastric maladies of the kind indicated yield to glycerin; he gives a drachm, a drachm and a half, and sometimes even two drachms, with a little of some simple bitter stomachic tincture, diluted to an ounce with water, thrice daily, between meals.—*New York Medical Journal.*

